# Quasicontinuous Cooperative Adsorption Mechanism in Crystalline Nanoporous Materials

**DOI:** 10.1021/acs.jpclett.2c01752

**Published:** 2022-07-25

**Authors:** Bartosz Mazur, Filip Formalik, Kornel Roztocki, Volodymyr Bon, Stefan Kaskel, Alexander V. Neimark, Lucyna Firlej, Bogdan Kuchta

**Affiliations:** † Department of Micro, Nano, and Bioprocess Engineering, Faculty of Chemistry, Wrocław University of Science and Technology, 50-370 Wrocław, Poland; ‡ Department of Chemical and Biological Engineering, Northwestern University, Evanston, Illinois 60208, United States; § Faculty of Chemistry, Adam Mickiewicz University, 61-614 Poznań, Poland; ∥ Chair of Inorganic Chemistry, Technische Universität Dresden, Bergstrasse 66, 01062 Dresden, Germany; ⊥ Department of Chemical and Biochemical Engineering, Rutgers University, Piscataway, New Jersey 08854, United States; # Laboratoire Charles Coulomb, University of Montpellier-CNRS, 34095 Montpellier, France; ∇ MADIREL, CNRS, Aix-Marseille University, 13397 Marseille, France

## Abstract

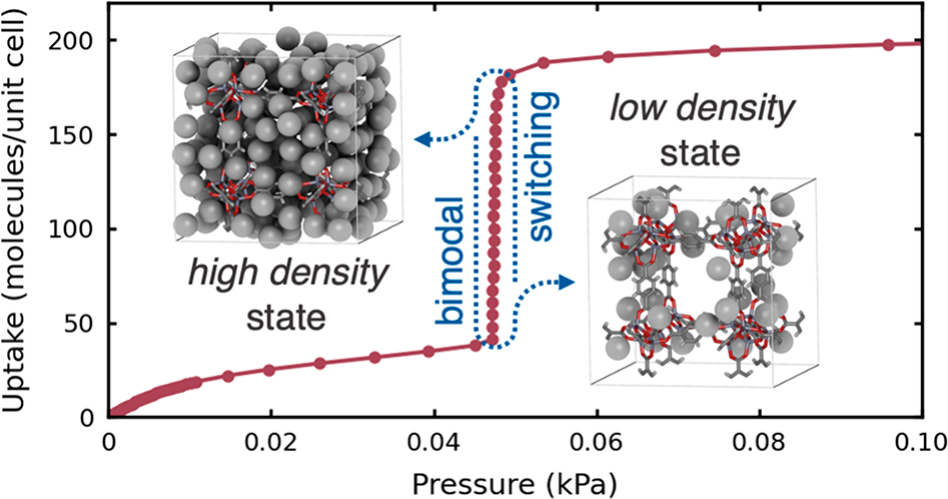

The hase behavior of confined fluids adsorbed in nanopores differs significantly from their bulk counterparts and depends on the chemical and structural properties of the confining structures. In general, phase transitions in nanoconfined fluids are reflected in stepwise adsorption isotherms with a pronounced hysteresis. Here, we show experimental evidence and an *in silico* interpretation of the reversible stepwise adsorption isotherm which is observed when methane is adsorbed in the rigid, crystalline metal–organic framework IRMOF-1 (MOF-5). In a very narrow range of pressures, the adsorbed fluid undergoes a structural and highly cooperative reconstruction and transition between low-density and high-density nanophases, as a result of the competition between the fluid–framework and fluid–fluid interactions. This mechanism evolves with temperature: below 110 K, a reversible stepwise isotherm is observed, which is a result of the bimodal distribution of the coexisting nanophases. This temperature may be considered as a critical temperature of methane confined to nanopores of IRMOF-1. Above 110 K, as the entropy contribution increases, the isotherm shape transforms to a common continuous *S-shaped* form that is characteristic to a gradual densification of the adsorbed phase as the pressure increases.

Nanoporous materials, such as metal–organic frameworks (MOFs), are widely explored for various practical applications from gas separations and storage to water harvesting and drug delivery.^1,2^ The engineering properties of MOFs are determined by the specifics of phase behavior of fluids confined in nanopores. It is well established that confined systems in general, and fluids adsorbed in nanopores in particular, have properties distinctly different from those of their bulk analogues.^3−6^ In particular, the critical conditions and the temperatures and pressures of vapor–liquid condensation and freezing–melting transitions are systematically shifted with respect to the bulk properties.^5^ Moreover, nanophase transitions generally exhibit pronounced hysteresis. Adsorption in nanoporous solids may lead to the structures not existing without the confining environment^3^ and to new types of phase transitions reflected in the singularities on the adsorption isotherm.

Recent numerical studies have suggested that a steep increase (in a very narrow pressure range) of the methane uptake in the rigid IRMOF-1^7^ framework may result from structural transformation occurring within adsorbate.^3^ A similar shape of the adsorption isotherm was also observed (both experimentally and numerically) in the case of CO_2_ adsorption in the same framework.^8^

In this work, we revisit the methane adsorption mechanism in IRMOF-1 and its interpretation, both experimentally and numerically. Using molecular modeling, we show that the *steplike* shape of the isotherms reflects the adsorbate structural transformation, induced by rapid and strongly cooperative adsorption, resulting from (i) competition between the fluid–framework and fluid–fluid interactions and (ii) the nanosized confining environment. We have shown the coexistence of two states of adsorbate of different densities between which the system fluctuates. This coexistence is observed only at low temperatures, and in an extremely narrow range of pressures. Such bistability of the adsorbate manifests itself as a step on the adsorption isotherm, observed both in molecular simulations and in experiment. Detailed information on the system, interaction models, simulation details, and experimental conditions is provided in the Supporting Information (SI).

The experimental and simulated isotherms of methane adsorption in IRMOF-1 at *T* = 92, 102, and 110 K (the range of vapor–liquid coexistence in bulk methane between the triple point and normal boiling temperatures of 90.7 and 111.65 K, respectively^9^) are presented in Figure 1 (left column). At low temperatures, they show well-defined discontinuity (vertical step) which disappears at the highest temperature. Such a shape of the adsorption isotherm usually suggests the occurrence of capillary condensation [type IV(b) isotherm according to the IUPAC classification^10^]. However, IRMOF-1 is a microporous material with two types of pores of diameter ∼1.5 and ∼1.1 nm^7^ (Figure S7). It is commonly assumed that adsorption in micropores proceeds through a gradual filling of the pore volume, and capillary condensation is not expected here.

**Figure 1:**
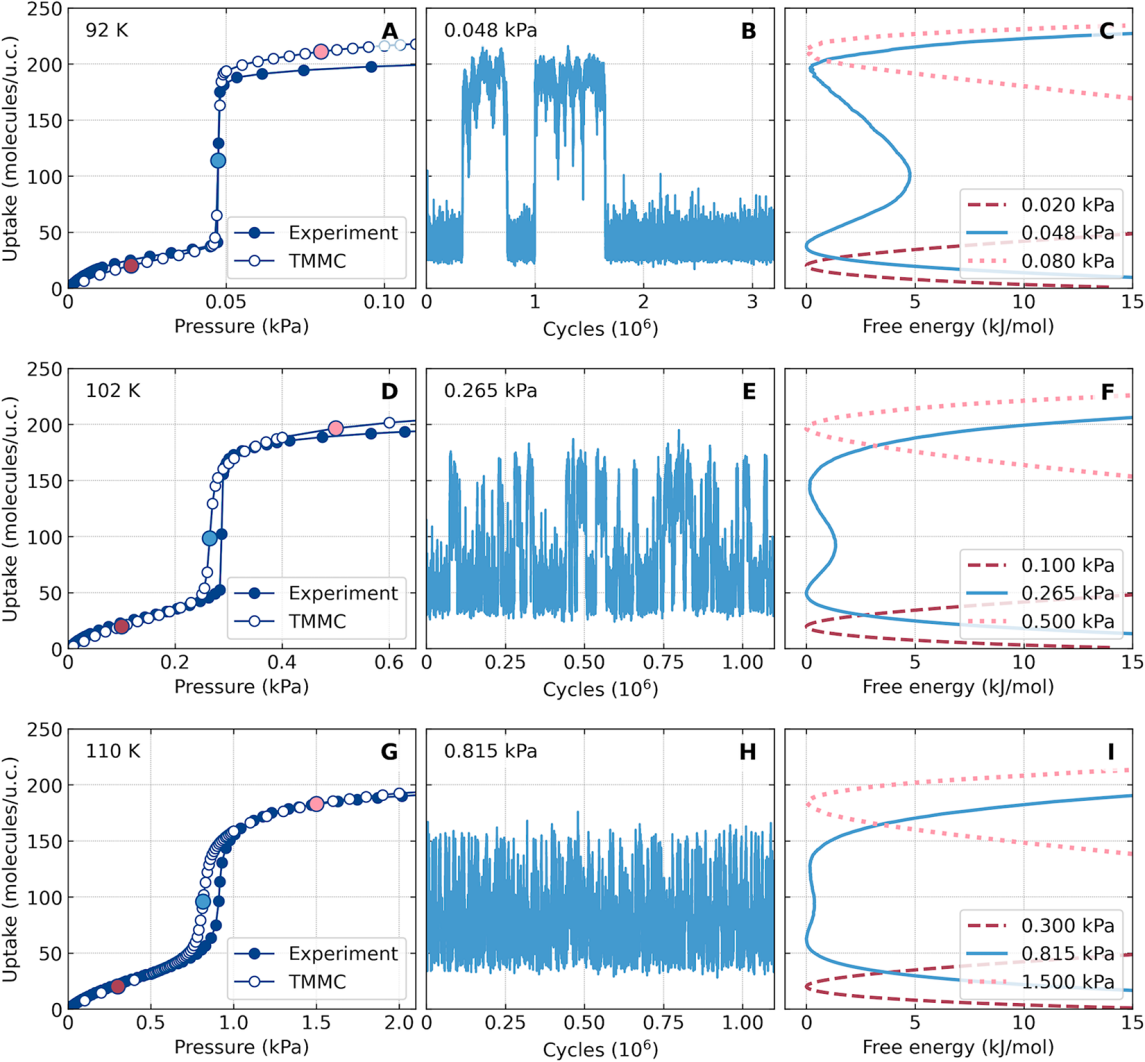
Left column: experimental and simulated, using transition matrix Monte Carlo (TMMC) simulations, isotherms of methane adsorption in IRMOF-1 at 92 K (A), 102 K (D), and 110 K (G). Middle column: fluctuations in the number of adsorbed methane molecules at 92 K (B), 102 K (E), and 110 K (H). Right column: free energy profiles for three points of the simulated (TMMC) isotherms—before, in the middle of, and after the adsorption step. The points are indicated on the isotherms with the corresponding colors. Note that the vertical axis is shared by all graphs in the row. The error bars are small and practically not visible (Figure S2), except for the transition range. The pressure region where the step is observed depends on temperature: 0.048 ± 0.003 kPa (at *T* = 92 K), 0.270 ± 0.01 kPa (at *T* = 102 K), and 0.830 ± 0.07 kPa (at *T* = 110 K). See the SI (Figure S3) for additional temperatures and trends.

To explain the mechanism of such *steplike* adsorption, we have carried out very long (about 3 million cycles) grand canonical Monte Carlo simulations in the *step* pressure range. We observed that the system behaves in a bimodal way: the adsorbate jumps dynamically between two states of different densities: *low density* (ld) (40–50 molecules/unit cell, structure of a partial monolayer) and *high density* (hd) (170–180 molecules/unit cell, pore filled) (Figure 1, middle column). The ld and hd states must be separated by an energy barrier which allows the nanostates to coexist. Such a situation is only possible at the nanoscale, where the relative amplitudes of fluctuations are higher than in the bulk (macroscopic systems),^11^ and the separation of states is energetically not favorable. Macroscopically, both states are undiscernible because the experimentally measured (therefore, averaged over time and sample volume) adsorption is a weighted sum of the ld and hd instantaneous uptakes, with the weights proportional to the time that the system spends in each state. At a lower temperature (92 K), the bimodal behavior is very pronounced: the time between bimodal switching is long (Figure 1B), and the jumping may not be observed during simulations of the same length. Of course, this limiting condition does not apply to the experimental measurements that are equilibrated for several minutes or even hours. At higher temperatures, the bimodal behavior transforms into a large amplitude fluctuation around the average uptake value, which indirectly indicates that the free energy barrier between the ld and hd states vanishes (Figure 1H).^11^

The frequency of transitions between the ld and hd states is determined by the height of the energy barrier, *E*_b_, and is proportional to exp(*−E*_b_/*k*_B_*T*). This estimate predicts that, for temperatures 92 K (*E*_b_ ∼ 6*k*_B_*T*), 102 K (*E*_b_ ∼ 2*k*_B_*T*), and 110 K (*E*_b_ ∼ 1*k*_B_*T*), the transition frequencies should follow the proportion 1:50:150 that roughly corresponds to the number of *jumps* observed in the respective MC simulations (Figure 1, middle column). This estimate validates our hypothesis about the adsorption mechanism.

To visualize and justify this statement, we calculated free energy profiles using grand canonical transition matrix MC (GC-TMMC) simulations.^12−14^ The results presented in Figure 1 (right column) demonstrate the presence of an energy barrier between the ld and hd states. The height of this barrier decreases with increasing temperature. It causes a change of the isotherm shape, from *steplike* to more continuous, *S-shaped*, with a lower slope in the transition region. To better understand the mechanism of this transition, we plotted the maps of adsorbate energy as a function of gas pressure and number of adsorbed molecules (Figure 2). At 92 K in the *jump* region, there are two minima separated by an energy barrier of the high state of ∼6*k*_B_*T* (see also Figure 1C). The *jump* itself is vertical because the system is fluctuating between low- and high-density states. At 110 K, the barrier is much lower, on the order of the thermal energy *k*_B_*T*, and the distribution of states (between the ld and hd states) evolves from bimodal to more continuous.

**Figure 2:**
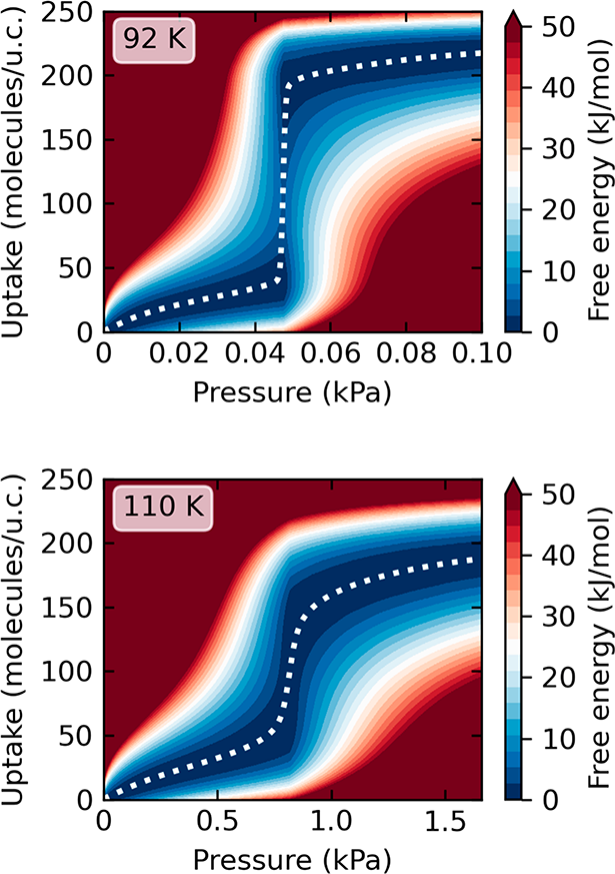
Free energy map as a function of pressure and number of adsorbed molecules at 92 K (top) and 110 K (bottom). See also Figure S6 in the SI annex. The white dotted lines represent the equilibrium isotherms (also called “net” isotherms^15^).

The model proposed above also suggests that the experimentally observed isotherm could be quasicontinuous if it was measured with a sufficiently small increase in pressure (*ΔP*). Figure 3 presents the experimental adsorption isotherms measured with step *ΔP* ∼ 0.3 Pa, much smaller than *ΔP* ∼ 40 Pa used in Figure 1. The step on the isotherm is now densely covered by the measured uptakes; however, at low temperatures (*T* < 110 K) the transition between the ld and hd states still appears to be almost vertical. This result confirms our hypothesis that, at low temperatures, the steplike isotherm results from the statistical average between ld and hd states distributed over the sample. The isotherm continuously evolves into an S-shaped form only when the temperature increases: the energy barrier between the ld and hd states decreases and becomes comparable with the energy of thermal motion of the adsorbate molecules. Such conditions were not at all explored in the paper by Fairen-Jimenez et al.^16^ which focused on the transition from S-shaped type V isotherm observed at 150 K to the type I isotherm at 300 K. The authors showed that the type V behavior observed at lower temperatures results from relatively weak methane–IRMOF-1 interactions. The increase in temperature is sufficient to shift the balance between fluid–solid and fluid–fluid interactions and to induce a transition from type V to type I behavior, characteristic for microporous materials.^10,17^

**Figure 3:**
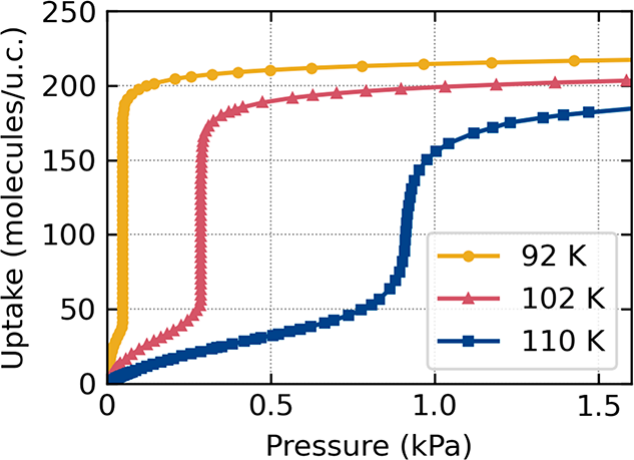
Experimental isotherms of methane adsorption in IRMOF-1 measured with the pressure step *ΔP* ∼ 0.3 Pa (for comparison, see Figure 1A,D,G, where *ΔP* ∼ 40 Pa).

Another important feature of methane adsorption in IRMOF-1 is its cooperative nature. Figure 4 (top, full symbols) shows the decomposition of the total adsorption isotherms into the isotherms calculated separately for the large and small pores^18−20^ of the IRMOF-1 structure (see Figure S7). Clearly, adsorption starts in the large pores, as the primary strong adsorption sites are located there (Figure 4, bottom). Similar behavior was observed when adsorption was selectively restricted in simulations to only large or small pores (Figure 4, top, open symbols). When uptake increases, the methane–methane interactions become stronger than those of methane–IRMOF-1. This causes a rearrangement of the adsorbed fluid and a complete filling of the pores. At the same time, the system becomes more stable: the average energy of adsorption, calculated as a sum of fluid–framework and fluid–fluid potential energy in the systems with a fixed number of molecules, decreases (Figure S4). The decomposition of the total adsorption isotherm suggests that the ld state corresponds to the adsorption at the high-energy sites in large pores, and the transition to the hd state occurs by simultaneous filling of large and small pores. Because adsorption in small pores in the ld state is negligible, it is prudent to conclude that the initial filling of large pores triggers the filling of small pores due to additional fluid–fluid interactions. The evolution of the adsorbate structure upon adsorption was monitored using methane density maps (see Figure S8). Although the distribution of adsorbed methane density substantially changes during the adsorption process, its symmetry is determined by the symmetry of the sorbent. We hypothesize that adsorbent symmetry may be crucial for the specific mechanism of steplike adsorption. For example, our results at 110 K show that the temperature disorder of the adsorbate already makes the mechanism more continuous. However, to confirm this conclusion, more extensive study is necessary.

**Figure 4:**
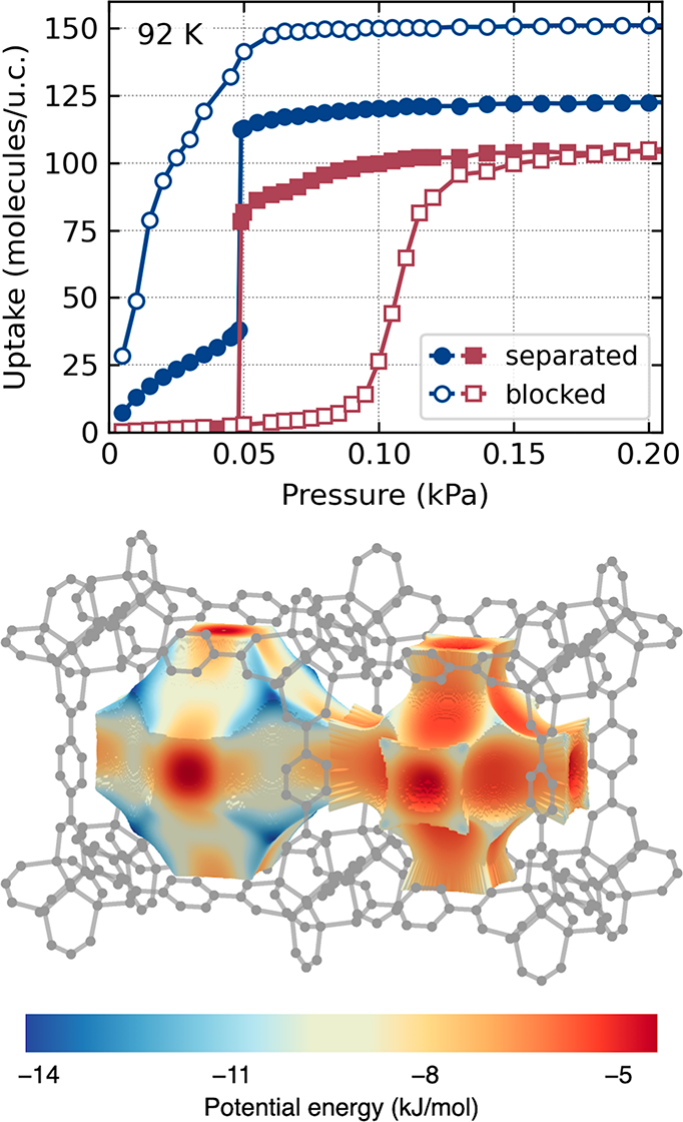
Top: isotherm of methane adsorption in IRMOF-1 at 92 K. Full symbols: adsorption observed only in the small (red squares) and only in the large (blue circles) pores. Open symbols: adsorption calculated by restricting fluid access to only small (red squares) or to only large (blue circles) pores. See also Figure S5 in the SI annex. Bottom: distribution of the methane adsorption energy in IRMOF-1. See also Figure S7 in the SI annex.

In conclusion, a new mechanism of adsorption, characterized by a steplike increase of the amount adsorbed in a very narrow range of pressures, was observed both numerically and experimentally in ordered nanoporous crystals of IRMOF-1. The existence in the transition range of two states of adsorbate of low (ld) and high (hd) density was corroborated by numerical simulations. The two states are separated by a small, temperature-dependent energy barrier, which allows the system to be in bimodal equilibrium, that is, dynamically jump between hd and ld states. This situation is observed only in a very narrow range of pressures. To the best of our knowledge, such bimodal density fluctuations have never been observed in the context of any porous materials.

The energy barrier between ld and hd states increases with lowering the temperature. Ultimately, at very low temperatures, the dynamic transition between states will not be observed numerically (in the finite simulation time), even if it may still be observed experimentally. This is an example of the rare-event process in a double-well potential with a high barrier and requires a special simulation approach.^21^

It is worth emphasizing that this type of behavior may be observed only at the nanoscale where the macroscopic separation of the phases is not possible, and the hd and ld regions dynamically coexist under the same thermodynamic conditions.^11^ In other words, in small systems of finite volume, in which the concept of the thermodynamic limit is no longer valid, the interface between phases cannot exist because of the too high energy cost.

The transformation between ld and hd structures can also be analyzed from another, more adsorption-based point of view. The ld structure can be considered as a *contact layer*, its structure being defined by the distribution of the strongest adsorption sites and forming a *monolayer-like* system. On the other hand, the hd state, where the intra-adsorbate interaction plays the major (stabilizing) role, and the interaction with the confining walls is negligible, may be considered as a *3D cluster* type. This means that the rapid steplike adsorption reported in this paper *cannot* be categorized as the nanoanalogue of the bulk gas–liquid transition. This observation is important for further exploration and understanding of the adsorption-induced, in-pore transformation. For example, with increasing temperature, the described coexistence of states vanishes, and the transformation is truly continuous. Microscopically, this also means that when the thermal fluctuations of the adsorbate make its structure dynamically disordered, the density of accessible states will be high enough to facilitate continuous adsorption, in analogy to the capillary condensation observed in mesopores. However, this aspect requires more fundamental studies.

## Supporting Information Available

Details on simulation methodology used in this work, additional simulation results of adsorption isotherms, adsorption energy profiles, free energy maps, minimum energy surface, adsorption density maps, and details on synthesis and characterization of IRMOF-1 and gas adsorption measurements (PDF)
Transparent Peer Review report available (PDF)

## Notes

*Data Availability*. Modified RASPA code for GC-TMMC simulations and all exemplary simulation input files are available at https://github.com/b-mazur.
