# Supplementary material for: Quasicontinuous Cooperative Adsorption Mechanism in Crystalline Nanoporous Materials
Source: J Phys Chem Lett. 2022 Jul 25;13(30):6961–5. doi: 10.1021/acs.jpclett.2c01752 (PMC9358707; doi:10.1021/acs.jpclett.2c01752)
Supplement: Supplementary file 1 — jz2c01752_si_001.pdf [file jz2c01752_si_001.pdf]

SUPPORTING INFORMATION

Quasi-Continuous Cooperative Adsorption Mechanism in Crystalline Nano-Porous Materials

Bartosz Mazur[a], Filip Formalik[a,b], Kornel Roztocki[c], Volodymyr Bon[d], Stefan Kaskel[d], Alexander V. Neimark[e], Lucyna Firlej[a,f], Bogdan Kuchta[a,g]

[a] Department of Micro, Nano, and Bioprocess Engineering, Faculty of Chemistry, Wrocław University of Science and Technology, 50-370 Wrocław, Poland
[b] Department of Chemical and Biological Engineering, Northwestern University, Evanston, Illinois 60208, United States
[c] Faculty of Chemistry, Adam Mickiewicz University, Uniwersytetu Poznańskiego 8, 61-614 Poznań, Poland
[d] Chair of Inorganic Chemistry, Technische Universität Dresden, Bergstrasse 66, 01062 Dresden, Germany
[e] Department of Chemical and Biochemical Engineering, Rutgers University, New Jersey, USA
[f] Laboratoire Charles Coulomb, University of Montpellier-CNRS, 34095 Montpellier, France
[g] MADIREL, CNRS, Aix-Marseille University, 13397 Marseille, France

Table of contest

TABLE OF CONTEST 1
STRUCTURE MODEL AND FORCE FIELD 2
DETAILS OF GCMC SIMULATIONS 3
DETAILS OF TMMC SIMULATIONS (FLAT HISTOGRAM APPROACH) 3
SIMULATION ADSORPTION RESULTS 5
ADSORPTION ENERGY 6
ADSORPTION PORE CONTRIBUTION 7
FREE ENERGY MAPS 8
MINIMUM ENERGY SURFACE 9
ADSORPTION DENSITY MAPS 10
EXPERIMENTAL SETUP 11
REFERENCES 12

Structure model and force field

The crystal structure of IRMOF-1 was taken from the work of Eddaoudi et al.[1] (CIF structure is available in the RASPA[2] package). The framework was kept rigid during the simulations. The interactions between the IRMOF-1 structure and the adsorbate (methane) were modeled using the standard Lennard-Jones 6-12 model. The LJ parameters for the MOF structure (listed in **Table S1**) were obtained from the DREIDING[3] force field. The methane molecule was modelled as a united atom using DACNIS force field. We increased the σ parameter for hydrogen (from 2.8464 Å to 3.195 Å) and decreased for methane (from 3.72 Å to 3.65 Å) to better reproduce experimental adsorption isotherms (**Figure S1A**). This modification is physically justified because (i) C–H bond length obtained from PXRD is shorter than the experimental[4], and (ii) at low temperatures, the methane molecule is effectively smaller due to the hindered rotation. We have checked the vapor-liquid equilibrium (VLE) for the presented model (**Figure S1B**). The difference in the density of the liquid phase results in an increase in the maximal uptake at higher pressure, which is understandable and acceptable in our case, because we are focused on investigating the adsorption mechanism and not the absolute value of the maximal adsorbed amount. All interactions were shifted to zero at a cutoff value of 12.0 Å. Lorentz–Berthelot mixing rules were applied for the MOF–adsorbate cross terms. The fugacity coefficient was calculated using the Peng-Robinson equation of state, implemented in the RASPA package.

Table S1. Lennard-Jones parameters used in GCMC simulations.

Atom type	ε (K)	σ (Å)
Zn	27.6772	4.045
O	48.1584	3.033
C	47.8565	3.473
H	7.6490	3.195
CH_4	158.5000	3.650

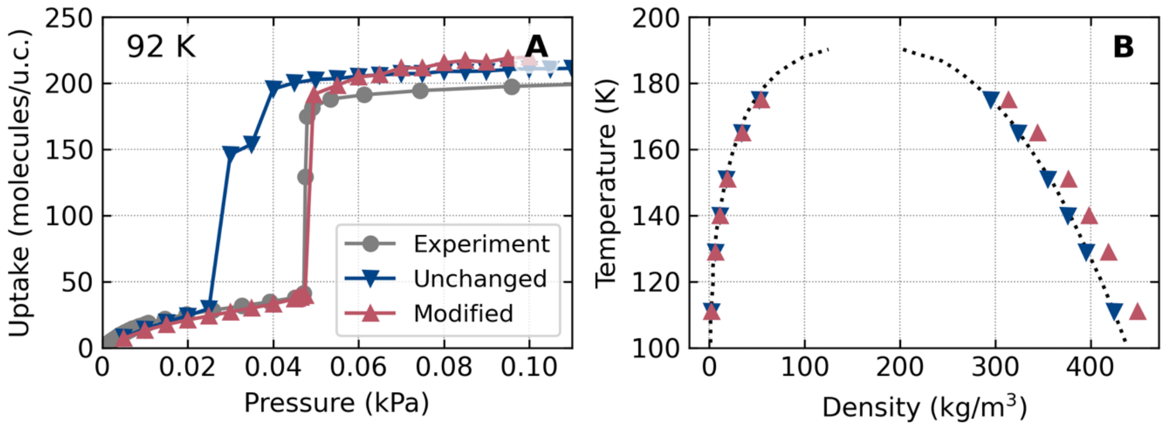

Figure S1. (A) Methane adsorption isotherms at 92 K, experimental (grey) and simulated with different σ parameter (blue and red for the original and modified force field, respectively). **(B)** Vapor–liquid coexistence curves for methane, experimental[5] (black dotted) and simulated with different σ parameter (colors correspond to the data in **A**).

Details of GCMC simulations

All GCMC simulations in this study were carried out using the RASPA[2] package. The system (simulation box) consisted of a single unit cell. The simulations contained at least 500 000 Monte Carlo cycles for both stabilization and production run. One cycle consists of N moves, where N is the number of adsorbed molecules at the beginning of the move (if there are fewer than 20 adsorbed molecules, then one cycle consists of 20 moves). The move type was chosen randomly between swap (insertion or deletion), reinsertion and translation. The probability of choosing a move was the same for all types of moves. If the system was not in equilibrium after a set number of cycles, the simulations were restarted for the same number of additional cycles until the system was fully equilibrated. An example of all input files used to run simulations using RASPA code are available at https://github.com/b-mazur.

Separated isotherms of adsorption (in the small and large IRMOF-1 pores) were calculated (post-processed) from methane positions recorded during simulation production runs. Blocking of the pores was achieved by restricting MC moves to the selected pore only.

Details of TMMC simulations (flat histogram approach)

To calculate the free energy of the system, we used a grand canonical transition matrix Monte Carlo (GC-TMMC) simulation – one of the flat histogram methods[6,7]. It is based on the computation of the density of states in biased Monte Carlo simulation (with non-Boltzmann distribution). The goal of such procedure is to sample states (characterized by some collective variable) with uniform probability and to calculate the unbiased acceptance rates for generating distributions of macrostate probabilities. To avoid biasing, we ran a parallel series of NVT simulations for all possible loading macrostates. Running individual simulations for each N artificially sets all probabilities of observing a given macrostates equal[8].

Consider a system at particular chemical potential μ (directly related to system pressure), temperature T and volume V. Such a system can be described with a grand canonical partition function $\Xi(\mu, V, \beta)$:

$$\Xi(\mu, V, \beta) = \sum_{N=0}^{\infty} e^{\beta,\mu,N} Q(N, V, \beta) \tag{1}$$

where $Q(N, V, \beta)$ is the canonical partition function and $\beta = (k_B T)^{-1}$. In this ensemble, the probability of observing a system containing a given number of N adsorbed particles is:

$$\Pi(N;\ \mu, V, \beta) = \frac{e^{\beta,\mu,N} Q(N, V, \beta)}{\Xi(\mu, V, \beta)} \tag{2}$$

This (statistical) value is directly related to the free energy of the system. In the Grand Canonical ensemble, the grand canonical free energy Ω (or potential of adsorption) is:

$$\Omega = F - \mu N \equiv -PV \tag{3}$$

As the grand canonical free energy is linked to the grand canonical partition function through the relation:

$$\Omega = -k_B T \ln \Xi(\mu, V, \beta) \tag{4}$$

we can calculate the difference of the grand canonical partition function of two states containing N_1 and N_2 particles:

$$\Delta\Omega_{1,2} = -k_B T \ln \langle \frac{\Pi(N_2;\ \mu VT)}{\Pi(N_1;\ \mu VT)} \rangle \tag{5}$$

In the GC-TMMC algorithm, standard moves, such as translation or reinsertion, are performed and accepted according to the conventional Metropolis criteria. Swap moves are replaced with moves consisting of ghost particle insertions and deletions, which are performed, but never accepted. However, the unbiased acceptance probabilities for such moves are calculated (as in GCMC simulations) and accumulated in the so-called C-matrix using Equations 6–9:

For insertion moves:

$$C(N, N+1) = C(N, N+1) + acc(N \to N+1), \tag{6}$$

$$C(N, N) = C(N, N) + [1 - acc(N \to N+1)], \tag{7}$$

For deletion moves:

$$C(N, N-1) = C(N, N-1) + acc(N \to N-1), \tag{8}$$

$$C(N, N) = C(N, N) + [1 - acc(N \to N-1)]. \tag{9}$$

The $C(N, N)$ element stores the probabilities of rejection of both insertion and deletion trial moves. To obtain $\Pi(N;\ \mu, V, T)$ from C-matrix the following balance is used:

$$P(N \to N+1)\Pi(N;\ \mu VT) = P(N+1 \to N)\Pi(N+1;\ \mu VT). \tag{10}$$

Equation 10 states that the overall probability P of a system initially in the state with N molecules to transit to state with $N + 1$ molecules must be equal to the overall probability P of a system with $N + 1$ molecules to transit to state with N molecules. It is a natural consequence of the microscopic reversibility of conventional MC algorithms. The macrostate transition probability is obtained directly from the C-matrix as

$$P(N \to N+1) = \frac{C(N, N+1)}{\sum_{\Delta \in \{-1,0,1\}} C(N+\Delta)}. \tag{11}$$

Using the described scheme, one can obtain the ratio of any two consecutive values of $\Pi(N;\ \mu, V, T)$. Therefore, all $\Pi(N;\ \mu, V, T)$ values can be expressed using a specific macrostate probability $\Pi(N = 0;\ \mu, V, T)$. Finally, the equilibrium value of loading $\langle N \rangle$ is determined as weighted average over all macrostate probabilities using:

$$\langle N \rangle = \frac{\sum_{N=0}^{N_{max}} N \cdot \Pi(N;\ \mu VT)}{\sum_{N=0}^{N_{max}} \Pi(N;\ \mu VT)} \quad (12)$$

where the factor of $\Pi(N = 0;\ \mu, V, T)$ cancels out.

The main advantage of the described flat histogram Monte Carlo method is the possibility to use the data from calculations at some particular chemical potential value to predict macrostate probability at any other value of chemical potential, using data reweighting[9]:

$$\ln \Pi(N;\ \mu' VT) = \ln \Pi(N;\ \mu VT) + \beta(\mu' - \mu)N. \quad (13)$$

All TMMC simulations were performed using modified RASPA code available at https://github.com/b-mazur. The modification consists in the addition of a new type of move, *GhostSwapProbability,* which performs ghost particle insertion or deletion in the manner described previously. The acceptance probability of such moves is summed over the whole simulation run (after equilibration part summation restarts from the beginning) and printed out in the output file every certain number of steps that must be specified in the input file.

Simulation adsorption results

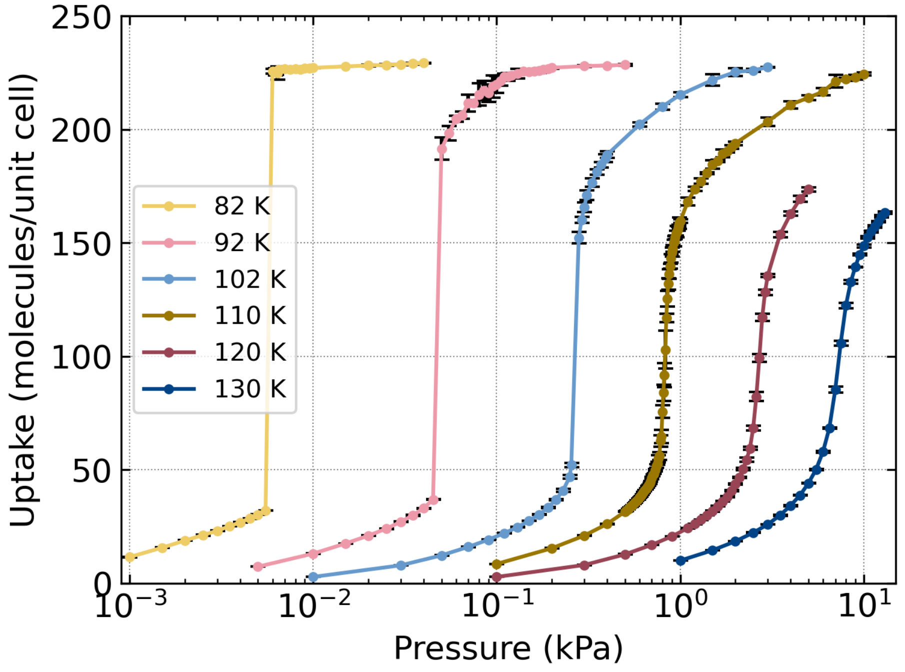

Figure S2. Simulated (Grand Canonical Monte Carlo) adsorption isotherms of CH_4 in IRMOF-1 at different temperatures. The error was computed by RASPA software by dividing the simulation in 5 blocks and calculating the standard deviation.

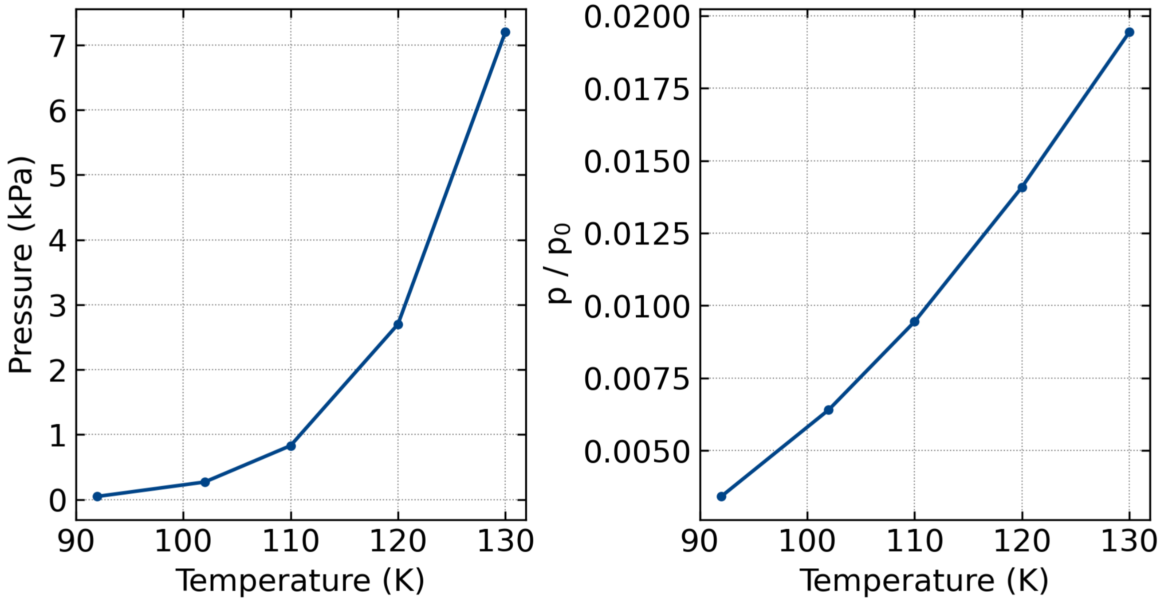

Figure S3. Pressure of transition as a function of temperature. Left: pressure in absolute scale, right: pressure in relative scale, p_0 values were calculated using Antoine equation[10].

Adsorption energy

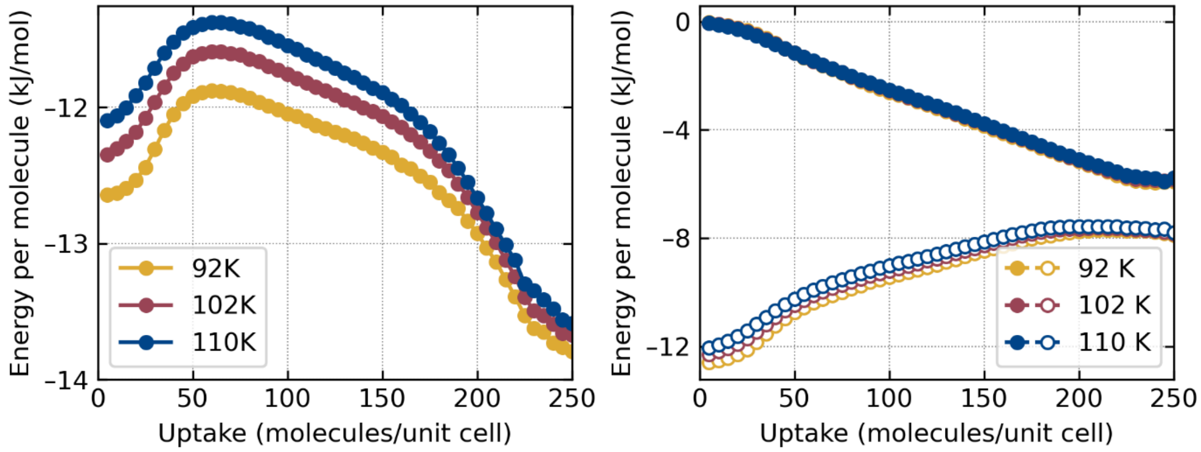

Figure S4. Average internal potential energy as a function of uptake (Canonical Monte Carlo). Left: total adsorption energy per molecule. Right: methane-MOF (open circles) and methane-methane potential energy (closed circles). The total energy is the sum of the methane-MOF and methane-methane contribution.

Adsorption pore contribution

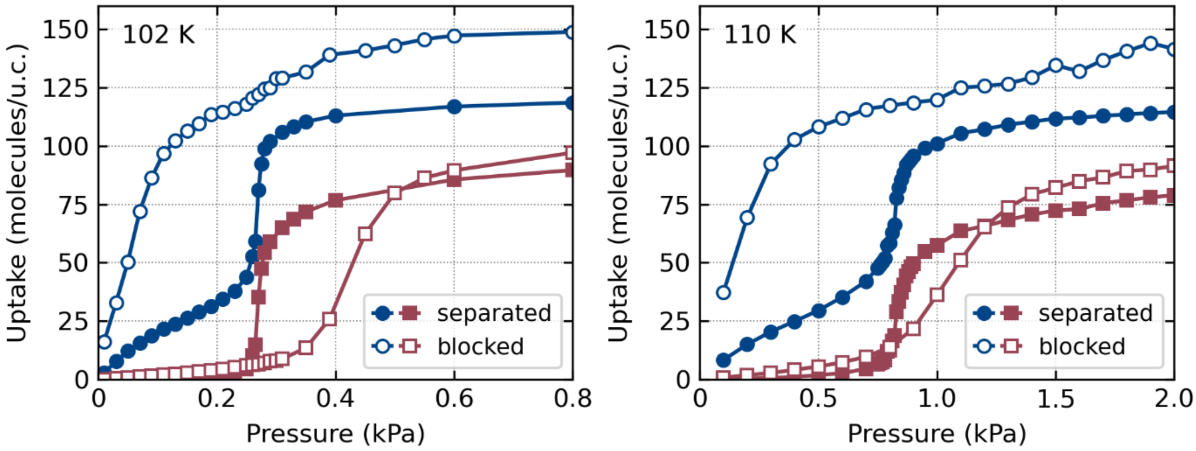

Figure S5. Isotherm of methane adsorption in IRMOF-1 at 102 (left) and 110 K (right). Full symbols: adsorption observed only in the small (red squares), and only in the large (blue circles) pores. Open symbols: adsorption calculated by restricting fluid access to only small (red squares) or to only large (blue circles) pores.

Free energy maps

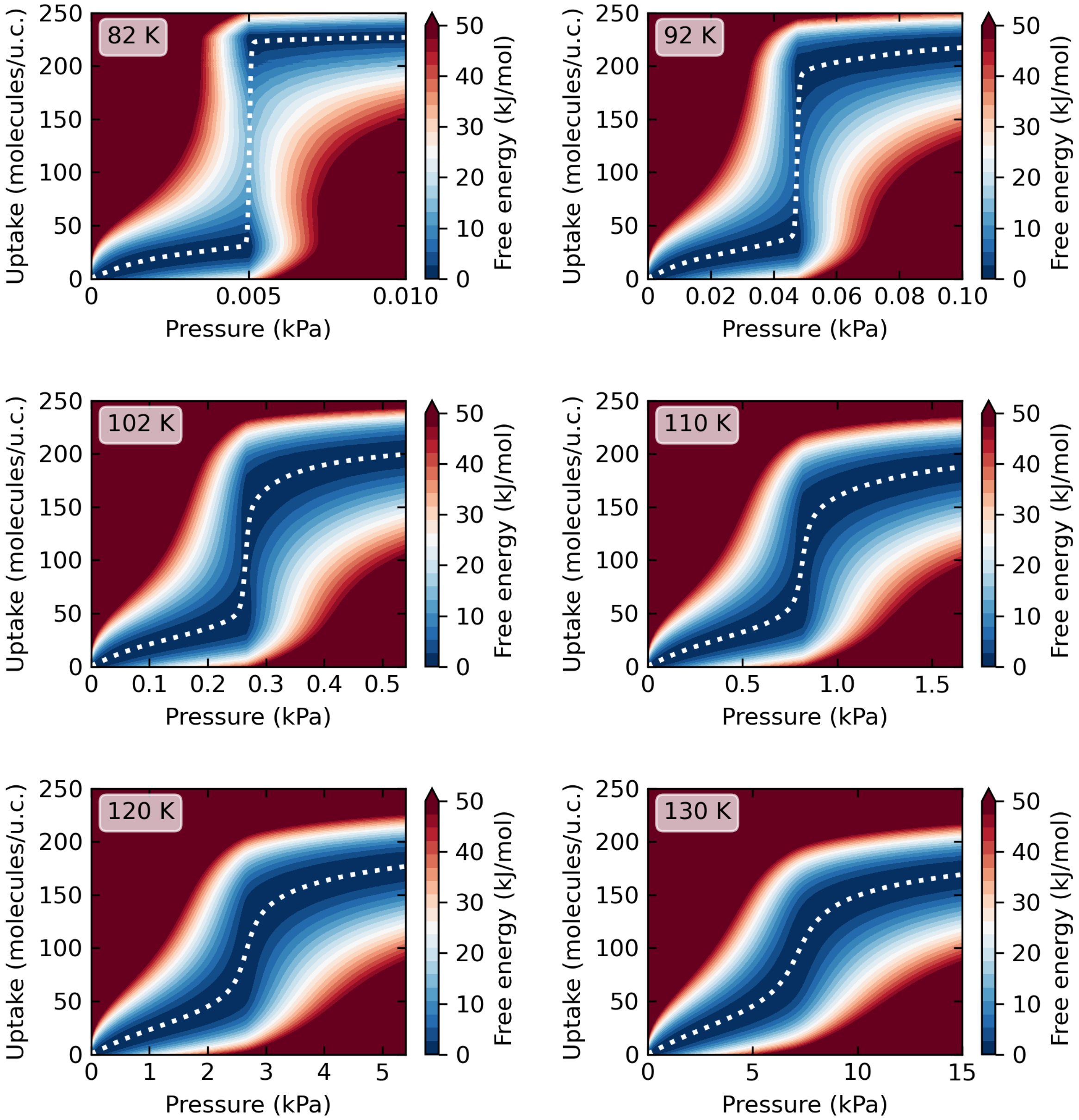

Figure S6. Free energy map as a function of pressure and number of adsorbed molecules at 82-130 K. The white dotted line represents the equilibrium isotherm (also referred as 'net isotherm'[11]) calculated with Equation 12.

Minimum energy surface

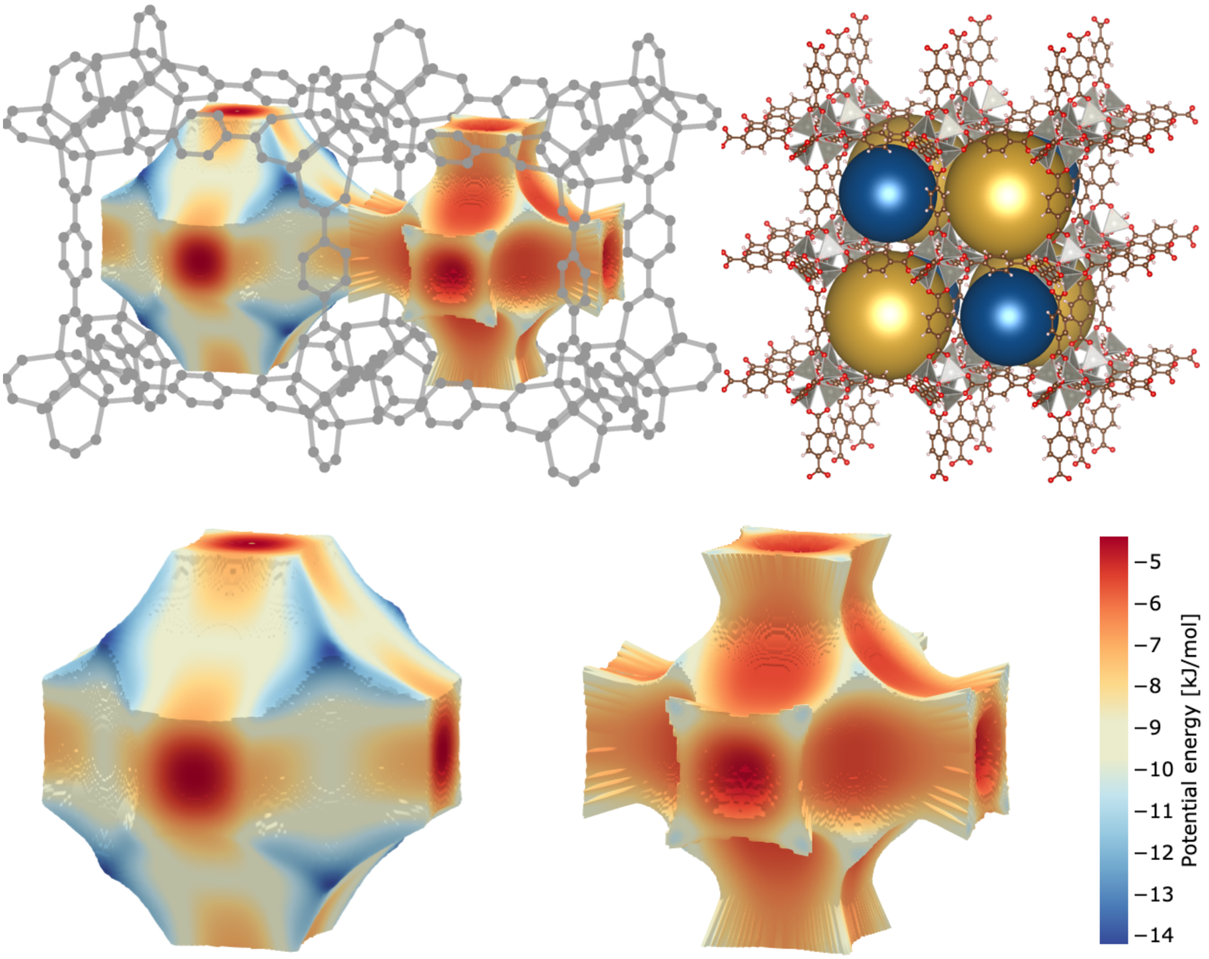

Figure S7. Distributions of the energies of the adsorbing sites on the surface of the two types of pores in the IRMOF-1 system (calculated for methane adsorption). The 3D profile represents the real in-pore surface shape. Top left: maps location inside IRMOF-1 structure (the left one is the larger one, shown with yellow sphere and the right one is the smaller one, shown with blue sphere on the right picture). Bottom: The energies of methane adsorption in the IRMOF-1 structure. The energies of the 3 most attractive adsorption sites are[12]: -1700 K, -1050 K (large pore), and -1200 K (small pore).

Adsorption density maps

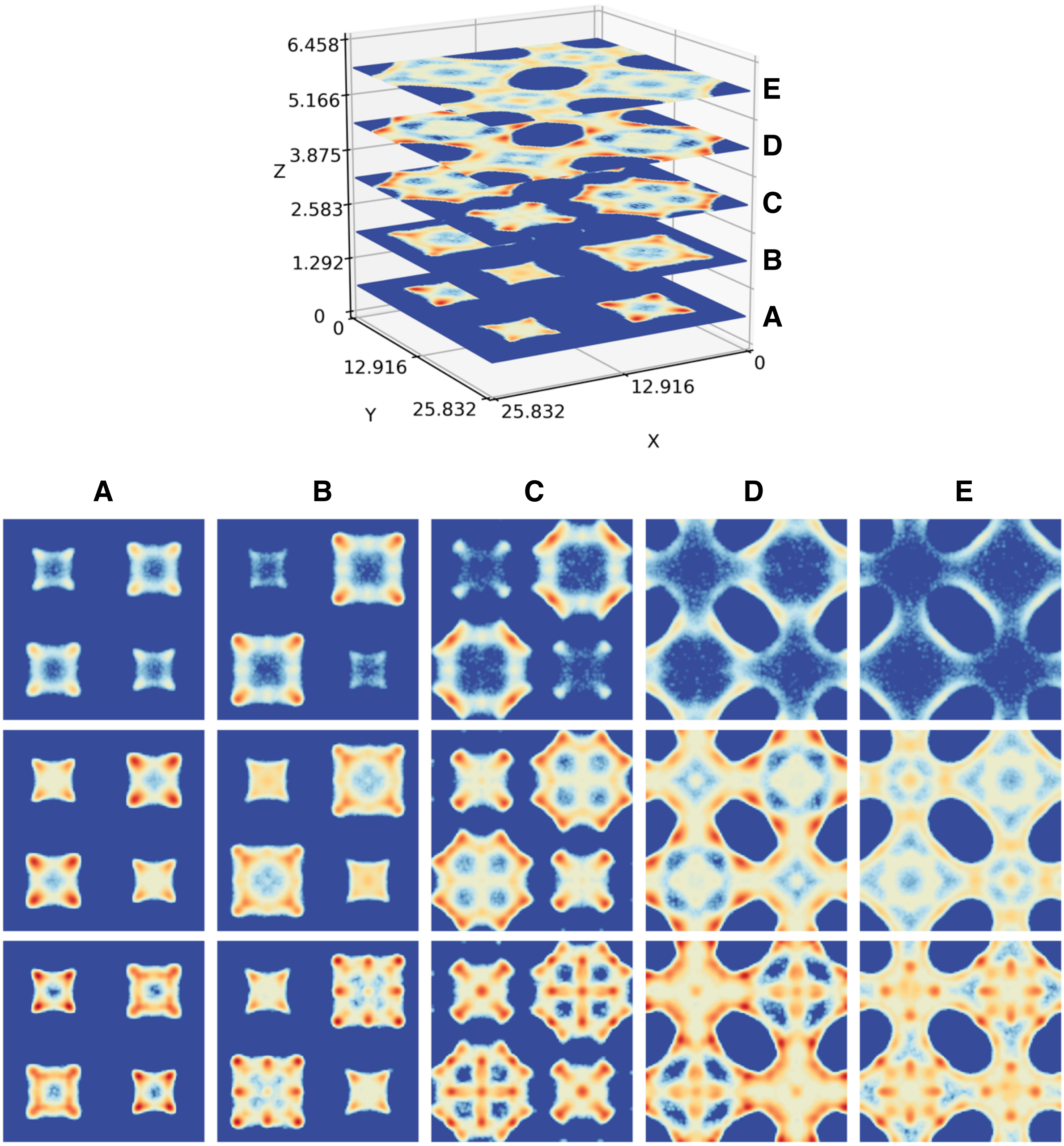

Figure S8. Top: slab location inside IRMOF-1 unit cell – note that for symmetry reasons, we represent only ¼ of the height of the unit cell. Bottom: simulated methane density maps in IRMOF-1 at 92 K. Each row corresponds to a selected point on the adsorption isotherm: top – before step on the isotherm (20 Pa, dark red point in Figure 1A, approximately 21 molecules/unit cell – GCMC simulation), middle – in the middle of it (light blue point in Figure 1A, forced configuration with constant number of 114 molecules/unit cell – CMC simulation), and bottom – after step (80 Pa, light red point in Figure 1A, approximately 215 molecules/unit cell – GCMC simulation). Large pores are on the bottom left and top right of the slabs. Red corresponds to the highest density, and blue corresponds to the lowest.

Experimental setup

Synthesis

IRMOF-1 was prepared in N,N-diethylformamide (DEF) solution according to the published method[13]. Due to the lack of product after 8 h postulated, the reaction time was extended to a total of 24 h. All other reagents and solvents were of analytical grade (Sigma Aldrich, Tokyo Chemical Industry) and were used without further purification.

Powder X-ray diffraction (PXRD) measurement

The PXRD pattern of the desolvated IRMOF-1 was measured at room temperature on a STOE STADI P diffractometer using Cu-Kα1 radiation (λ = 1.54059 Å) and a 1D detector (Mythen, Dectris). Measurements were performed in a transmission mode using a rotating flatbed sample holder, 2Θ steps of 6° and an exposition time of 20 s per step. Before measurement, IRMOF-1 was mounded in the glovebox and protected from air using a parafilm.

Gas adsorption measurements

Prior to the physisorption measurements, the as-synthesized IRMOF-1 was washed eight times with 20-30 mL of anhydrous DMF, each time letting the solid soak in DMF for 1-3 h. The DMF was decanted, and the sample was washed 7 times with 20-30 ml of anhydrous CH_2Cl_2, each time letting IRMOF-1 soak in dichloromethane for 1-3 h. Using the Schlenk technique, the excess solvent was removed in Ar flow and the remaining solid was desolvated under reduced pressure (~10^{-3} kPa) at 363 K for ~16 h. Then, the sample was transferred in the Schlenk tube into glovebox (MBRAUN).

The CH_4 (99.999% purity) adsorption studies were performed on a *BELSORP-max* adsorption apparatus (MicrotracBEL Corp.).

BELSORP-max was connected to the home-built adsorption cell, connected to the closed cycle helium cryostat DE-202AG (ARS). The adsorption temperature was set by temperature controller LS-336 (LAKE SHORE), and the heat produced by the cryostat was removed from the system by a water-cooled helium compressor ARS-2HW. 32.2 mg of desolvated IRMOF-1 was placed in an adsorption cell, sealed by a copper gasket from the exterior with a copper dome and insulated by dynamic vacuum ($p < 10^{-4}$ kPa), and connected to the *BELSORP-max* adsorption instrument with a 1/8 inch stainless steel capillary. After sealing the sample was degassed in a dynamic ultra-high vacuum of 0.01 Pa for 12 h. at 298 K. The pressure changes for 500 s within 1% of pressure were considered as adsorption equilibrium conditions.

Comment on measurement accuracy

The precision has been assured by the experimental conditions, that is, we used 32.2 mg of sample, the dead volume was 41.44 cm^3 and the surface area per adsorption cell was 121.2 m^2 (which is two times higher than recommended). Consequently, the shapes of the isotherms are smooth and without any outliers, which is the sign of temperature stability and sufficient amount of the sample in the cell.

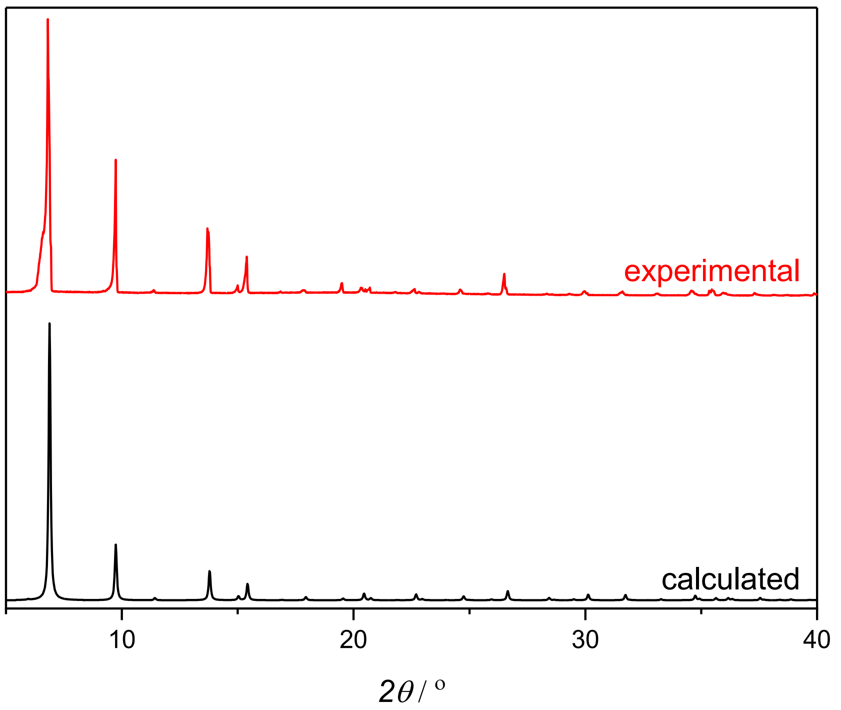

Figure S9. Comparison of PXRD patterns of desolvated IRMOF-1: calculated (CCDC 256965; database identifier: SAHYIK), based on SC-XRD measurements at 213 K (black); and experimental, measured for the polycrystalline sample in a transmission mode using a rotating flatbed sample holder at room temperature (red).

References

(1) Eddaoudi, M.; Kim, J.; Rosi, N.; Vodak, D.; Wachter, J.; O'Keeffe, M.; Yaghi, O. M. Systematic Design of Pore Size and Functionality in Isoreticular MOFs and Their Application in Methane Storage. *Science (1979)* **2002**, *295* (5554), 469–472. https://doi.org/10.1126/science.1067208.

(2) Dubbeldam, D.; Calero, S.; Ellis, D. E.; Snurr, R. Q. RASPA: Molecular Simulation Software for Adsorption and Diffusion in Flexible Nanoporous Materials. *Molecular Simulation* **2016**, *42* (2), 81–101. https://doi.org/10.1080/08927022.2015.1010082.

(3) Mayo, S. L.; Olafson, B. D.; Goddard, W. A. DREIDING: A Generic Force Field for Molecular Simulations. *The Journal of Physical Chemistry* **1990**, *94* (26), 8897–8909. https://doi.org/10.1021/j100389a010.

(4) Cooper, R. I.; Thompson, A. L.; Watkin, D. J. CRYSTALS Enhancements: Dealing with Hydrogen Atoms in Refinement. *Journal of Applied Crystallography* **2010**, *43* (5), 1100–1107. https://doi.org/10.1107/S0021889810025598.

(5) Martin, M. G.; Siepmann, J. I. Transferable Potentials for Phase Equilibria. 1. United-Atom Description of n-Alkanes. *The Journal of Physical Chemistry B* **1998**, *102* (14), 2569–2577. https://doi.org/10.1021/jp972543+.

(6) Wang, F.; Landau, D. P. Efficient, Multiple-Range Random Walk Algorithm to Calculate the Density of States. *Physical Review Letters* **2001**, *86* (10). https://doi.org/10.1103/PhysRevLett.86.2050.

(7) Wang, F.; Landau, D. P. Determining the Density of States for Classical Statistical Models: A Random Walk Algorithm to Produce a Flat Histogram. *Physical Review E - Statistical Physics, Plasmas, Fluids, and Related Interdisciplinary Topics* **2001**, *64* (5). https://doi.org/10.1103/PhysRevE.64.056101.

(8) Shen, V. K.; Errington, J. R. Determination of Fluid-Phase Behavior Using Transition-Matrix Monte Carlo: Binary Lennard-Jones Mixtures. *Journal of Chemical Physics* **2005**, *122* (6). https://doi.org/10.1063/1.1844372.
(9) Errington, J. R.; Shen, V. K. Direct Evaluation of Multicomponent Phase Equilibria Using Flat-Histogram Methods. *Journal of Chemical Physics* **2005**, *123* (16). https://doi.org/10.1063/1.2064628.
(10) Prydz, R.; Goodwin, R. D. Experimental Melting and Vapor Pressures of Methane. *The Journal of Chemical Thermodynamics* **1972**, *4* (1), 127–133. https://doi.org/10.1016/S0021-9614(72)80016-8.
(11) Shen, V. K.; Siderius, D. W.; Mahynski, N. A. Molecular Simulation of Capillary Phase Transitions in Flexible Porous Materials. *The Journal of Chemical Physics* **2018**, *148* (12), 124115. https://doi.org/10.1063/1.5022171.
(12) Dubbeldam, D.; Frost, H.; Walton, K. S.; Snurr, R. Q. Molecular Simulation of Adsorption Sites of Light Gases in the Metal-Organic Framework IRMOF-1. *Fluid Phase Equilibria* **2007**, *261* (1–2), 152–161. https://doi.org/10.1016/j.fluid.2007.07.042.
(13) Kaye, S. S.; Dailly, A.; Yaghi, O. M.; Long, J. R. Impact of Preparation and Handling on the Hydrogen Storage Properties of Zn_4O(1,4-Benzenedicarboxylate)$_3$ (MOF-5). *J Am Chem Soc* **2007**, *129* (46), 14176–14177. https://doi.org/10.1021/ja076877g.
