# Supplementary material for: Quasicontinuous Cooperative Adsorption Mechanism in Crystalline Nanoporous Materials
Source: J Phys Chem Lett. 2022 Jul 25;13(30):6961–5. doi: 10.1021/acs.jpclett.2c01752 (PMC9358707; doi:10.1021/acs.jpclett.2c01752)
Supplement: Supplementary file 2 — jz2c01752_si_002.pdf [file jz2c01752_si_002.pdf]

jz-2022-01752b.R1

Name: Peer Review Information for "Quasi-continuous Cooperative Adsorption Mechanism in Crystalline Nanoporous Materials"

First Round of Reviewer Comments

Reviewer: 1

Comments to the Author

This manuscript presents a systematic numerical approach to cooperative adsorption mechanism and its temperature dependence. The Monte Carlo simulation carried out by the authors appears to have been conducted with utmost care. They have identified the presence of the low density (ld) and high density (hd) states, characterized their nature and how they change with their temperature.

This manuscript contains a significant finding about the role of bimodal density fluctuations in porous materials and their role in the functional shape of adsorption isotherms.

This study seems to have been conducted with care and its quality is high. The writing is clear. The subject matter is of importance not only to adsorption specialists but also to anyone who is interested in surface science and confined fluids. The insights contained in the article is interesting and merits publication.

Reviewer: 2

Comments to the Author

The authors presented a combined experimental and computational study to discuss an interesting isotherm transformation of methane confined to a rigid MOF-5. Their results show that at low temperatures (< 110 K) and between a narrow pressure region, a new 'steplike' adsorption isotherm could exist. In addition, the new isotherm is characterized by the coexisting and the quasi-continuous transition of low-density and high-density adsorption states. The manuscript is well organized, with adequate details in the supporting information. I recommend this work be published in the Journal of Physical Chemistry Letters after the following comments have been addressed.

(1) Page 1, Paragraph 3, the author stated that "This co-existence is observed only at low temperatures, and in an extremely narrow range of pressures. Such bi-stability of the adsorbate manifests itself as a step on the adsorption isotherm,…". Please clearly define the pressure region where such a new adsorption mechanism holds. Also, please further discuss the importance of such co-existence. If the bi-stability exists under extremely special conditions, why this finding and related mechanism would matter to experiments or applications?

(2) For numerical calculations and experimental results (Figs. 1, 3, 4), please add and discuss the error bars. While the observed phenomenon is sensitive to temperature and pressure, the error bars could impact the observations significantly.

(3) Page 4, the paragraph under Fig. 4, "…, it is prudent to conclude that the filling of large pores triggers the filling of small pores due to strong fluid-fluid interactions." This statement is confusing and possibly incorrect. It makes sense that at lower densities, methane has a preferential residence in larger pores, which is due to methane-MOF interactions. With the increase of methane density, methane molecules would saturate the large pores and naturally start to occupy the small pores. I do not see the role of fluid-fluid interactions. Also, is methane-methane interaction stronger than methane-MOF interaction?

(4) Page 4, the paragraph under Fig. 4, "We hypothesize that adsorbent symmetry may be crucial for the specific mechanism of steplike adsorption,…" It is hard to understand this hypothesis. Can the authors clearly elaborate on this? Real MOF samples come with all sorts of defects, which will break down the symmetry. Do you still expect the reported observations in real MOFs or other asymmetric nanoporous materials?

(5) Page 4, Paragraph 3, "This is an example of the rare-event process in a double-well potential with a high barrier…" What do you mean by "a double-well potential"? Do the authors imply that the choice of force fields matters?

(6) Page 5, the last paragraph, "Microscopically, it also means that when the thermal fluctuations of the adsorbate make the structure dynamically disordered,…" What is the "structure", the structure of the first cluster/layer of adsorbates, or the structure of MOF?

Author's Response to Peer Review Comments:

Response in the attached file

Reviewer(s)' Comments to Author and responses:

Reviewer: 1

Recommendation: This paper represents a significant new contribution and should be published as is.

Comments:
This manuscript presents a systematic numerical approach to cooperative adsorption mechanism and its temperature dependence. The Monte Carlo simulation carried out by the authors appears to have been conducted with utmost care. They have identified the presence of the low density (ld) and high density (hd) states, characterized their nature and how they change with their temperature. This manuscript contains a significant finding about the role of bimodal density fluctuations in porous materials and their role in the functional shape of adsorption isotherms. This study seems to have been conducted with care and its quality is high. The writing is clear. The subject matter is of importance not only to adsorption specialists but also to anyone who is interested in surface science and confined fluids. The insights contained in the article is interesting and merits publication.

We appreciate the positive comments which enforce our conviction about the general interest and the importance of the presented phenomenon, especially in the field of general nanoscience and nano-thermodynamics.

Reviewer: 2

Recommendation: This paper is probably publishable, but major revision is needed; I do not need to see future revisions.

Comments:
The authors presented a combined experimental and computational study to discuss an interesting isotherm transformation of methane confined to a rigid MOF-5. Their results show that at low temperatures (< 110 K) and between a narrow pressure region, a new 'steplike' adsorption isotherm could exist. In addition, the new isotherm is characterized by the coexisting and the quasi-continuous transition of low-density and high-density adsorption states. The manuscript is well organized, with adequate details in the supporting information. I recommend this work be published in the Journal of Physical Chemistry Letters after the following comments have been addressed.

(1) Page 1, Paragraph 3, the author stated that "This co-existence is observed only at low temperatures, and in an extremely narrow range of pressures. Such bi-stability of the adsorbate manifests itself as a step on the adsorption isotherm,…". Please clearly define the pressure region where such a new adsorption mechanism holds. Also, please further discuss the importance of such co-existence. If the bi-stability exists under extremely special conditions, why this finding and related mechanism would matter to experiments or applications?

The pressure region where the step is observed depends on temperature: T = 92 K (0.047 $\pm$ 0.003 kPa), T = 102 K (0.285 $\pm$ 0.01 kPa), T = 110 K (0.915 $\pm$ 0.07 kPa). So, this region can be controlled by temperature. **We have added the information (Figure 1 caption and SI supplement: Figure S3).**

The importance of such coexistence has two aspects: (i) as a manifestation of nano-scale properties in adsorption and (ii) practical, in any storage, separation or detection type applications, because in the coexistence region the uptake is not defined macroscopically and very precisely defined outside this region.

(2) For numerical calculations and experimental results (Figs. 1, 3, 4), please add and discuss the error bars. While the observed phenomenon is sensitive to temperature and pressure, the error bars could impact the observations significantly.

The error bars are very small and practically not visible on the graph scale. The numerical precision has been achieved by the runs long enough to reduce the statistical uncertainty to negligible values. In experiment the precision has been assured by the experimental conditions, that is, we used 32.2 mg of sample, the dead volume was 41.44 cm^3 and the surface area per adsorption cell was 121.2 m^2 (which is two times higher than recommended). Consequently, the shapes of the isotherms are smooth without any outliers, which is the sign of temperature stability and sufficient amount of the sample in the cell.

We have added this information to the SI part (Figure S2). **Short information has been included in the Figure 1 caption.**

(3) Page 4, the paragraph under Fig. 4, "…, it is prudent to conclude that the filling of large pores triggers the filling of small pores due to strong fluid-fluid interactions." This statement is confusing and possibly incorrect. It makes sense that at lower densities, methane has a preferential residence in larger pores, which is due to methane-MOF interactions. With the increase of methane density, methane molecules would saturate the large pores and naturally start to occupy the small pores. I do not see the role of fluid-fluid interactions. Also, is methane-methane interaction stronger than methane-MOF interaction?

Our conclusion suggests that the initial adoption due to the strongest adsorption sites in the large pore triggers adsorption in the small pores due to the methane-methane interaction, that is, the adsorption in the small pore happens at lower pressure (as compared to the blocked, separate pore, see Figure 4) because of the additional attractive adsorption with the methane molecules already adsorbed in large pore. **We have slightly modified the initial text to emphasize the influence of ADDITIONAL fluid-fluid interaction.** The effect of interaction has been already described in the same paragraph.

The methane-methane interaction is definitely stronger than the methene-(any MOF atom) interaction (see Table S1). It means that the accumulative interaction of a methane molecule with a group of methane molecules can be stronger that the methane-MOF interaction. This effect is the main reason for the adsorbate structural changes when the density of methane molecules in pores increases. **This information already exists in the same paragraph.**

(4) Page 4, the paragraph under Fig. 4, "We hypothesize that adsorbent symmetry may be crucial for the specific mechanism of steplike adsorption,…" It is hard to understand this hypothesis. Can the authors clearly elaborate on this? Real MOF samples come with all sorts of defects, which will break down the symmetry. Do you still expect the reported observations in real MOFs or other asymmetric nanoporous materials?

Distributions of adsorption sites follow the high of the pore requires. All equivalent (by symmetry) sites are filled at the same time. As a consequence, the adsorption uptake increases in a non-continuous way. This is the logical and theoretical justification of our hypothesis. Even real MOFs, at low temperature, will preserve most of the symmetry properties if the concentration of the defects is not high. **As we mentioned in the manuscript, to confirm this conclusion more extensive study is necessary to verify this hypothesis**.

If the symmetry is reduced (because of defects introduced during the synthesis, or by increased disorder (of adsorbed molecules) at higher temperature, new uptake quantities can be stabilized and the bistability may disappear. **For example, our results at 110 K shows that the temperature disorder of the adsorbate makes already the mechanism more classical (this argument has been inserted into the revised version),** that is, the barrier between the low-density and high-density states practically is not present anymore and the mechanism switches from "quasi-continuous" into real continuous behavior

(5) Page 4, Paragraph 3, "This is an example of the rare-event process in a double-well potential with a high barrier…" What do you mean by "a double-well potential"? Do the authors imply that the choice of force fields matters?

The notion of rare-events, that is fluctuations having low probability (in this case, fluctuation over the barrier of a double well potential, as we show in the Figure 1) **has been discussed in the reference 21** (Tuckerman, M. Statistical Mechanics: Theory and Molecular Simulation; Oxford university press, 2010).

(6) Page 5, the last paragraph, "Microscopically, it also means that when the thermal fluctuations of the adsorbate make the structure dynamically disordered,…" What is the "structure", the structure of the first cluster/layer of adsorbates, or the structure of MOF?

The structure of the adsorbate, which affects the mechanism of adsorption. We have specified in the text which structure we refer to. **This comment is related to comments (4) above**, that is, to the decrease of the symmetry with increasing temperature.
